# Cardiac Sympathetic Denervation in 6-OHDA-Treated Nonhuman Primates

**DOI:** 10.1371/journal.pone.0104850

**Published:** 2014-08-18

**Authors:** Valerie Joers, Kristine Dilley, Shahrose Rahman, Corinne Jones, Jeanette Shultz, Heather Simmons, Marina E. Emborg

**Affiliations:** 1 Preclinical Parkinson's Research Program, Wisconsin National Primate Research Center, University of Wisconsin-Madison, Madison, Wisconsin, United States of America; 2 Neuroscience Training Program, University of Wisconsin-Madison, Madison, Wisconsin, United States of America; 3 Communication Sciences and Disorders Training Program, University of Wisconsin-Madison, Madison, Wisconsin, United States of America; 4 Cellular and Molecular Pathology Training Program, University of Wisconsin-Madison, Madison, Wisconsin, United States of America; 5 Department of Medical Physics, University of Wisconsin-Madison, Madison, Wisconsin, United States of America; Emory University, United States of America

## Abstract

Cardiac sympathetic neurodegeneration and dysautonomia affect patients with sporadic and familial Parkinson's disease (PD) and are currently proposed as prodromal signs of PD. We have recently developed a nonhuman primate model of cardiac dysautonomia by iv 6-hydroxydopamine (6-OHDA). Our *in vivo* findings included decreased cardiac uptake of a sympathetic radioligand and circulating catecholamines; here we report the postmortem characterization of the model. Ten adult rhesus monkeys (5–17 yrs old) were used in this study. Five animals received 6-OHDA (50 mg/kg iv) and five were age-matched controls. Three months post-neurotoxin the animals were euthanized; hearts and adrenal glands were processed for immunohistochemistry. Quantification of immunoreactivity (ir) of stainings was performed by an investigator blind to the treatment group using NIH ImageJ software (for cardiac bundles and adrenals, area above threshold and optical density) and MBF StereoInvestigator (for cardiac fibers, area fraction fractionator probe). Sympathetic cardiac nerve bundle analysis and fiber area density showed a significant reduction in global cardiac tyrosine hydroxylase-ir (TH; catecholaminergic marker) in 6-OHDA animals compared to controls. Quantification of protein gene protein 9.5 (pan-neuronal marker) positive cardiac fibers showed a significant deficit in 6-OHDA monkeys compared to controls and correlated with TH-ir fiber area. Semi-quantitative evaluation of human leukocyte antigen-ir (inflammatory marker) and nitrotyrosine-ir (oxidative stress marker) did not show significant changes 3 months post-neurotoxin. Cardiac nerve bundle α-synuclein-ir (presynaptic protein) was reduced (trend) in 6-OHDA treated monkeys; insoluble proteinase-K resistant α-synuclein (typical of PD pathology) was not observed. In the adrenal medulla, 6-OHDA monkeys had significantly reduced TH-ir and aminoacid decarboxylase-ir. Our results confirm that systemic 6-OHDA dosing to nonhuman primates induces cardiac sympathetic neurodegeneration and loss of catecholaminergic enzymes in the adrenal medulla, and suggests that this model can be used as a platform to evaluate disease-modifying strategies aiming to induce peripheral neuroprotection.

## Introduction

Cardiac sympathetic neurodegeneration and dysautonomia affect sporadic and familial cases of Parkinson's disease (PD) [1], [2], [3], [4], and are currently proposed to predate PD motor syndrome [5], [6], [7], [8], [9]. Orthostatic hypotension, fatigue, and abnormal control of electrical heart activity are typical manifestations of these pathologies. Reduced circulating catecholamines can also be present and is more commonly found in PD patients exhibiting orthostatic hypotension [10], [11], [12].

Loss of cardiac sympathetic function in PD has been demonstrated *in vivo* with single photon emission computed tomography (SPECT) and positron emission tomography (PET) imaging (reviewed in [13]). Neuropathological studies have confirmed cardiac nerve loss showing reduced tyrosine hydroxylase (TH) and neurofilament immunostaining in epicardial nerve bundles [14], [15], [16]. In addition, Lewy bodies (intracytoplasmic inclusions composed mainly of aggregated α-synuclein), which are a classical pathological feature of PD, have been described in cardiac nerve structures including cardiac plexus [17], cardiac ganglia within the interatrial groove, epicardial bundles and nerves that innervate the myocardium, as well as in the adrenal medulla [18], [19].

Cardiac sympathetic neurodegeneration and dysautonomia symptoms can seriously impact the quality of life of PD patients, as they do not respond to classical anti-parkinsonian medications and can even be worsened by them [20]. Well-characterized animal models in which to study these pathologies and test disease-modifying strategies that target the peripheral sympathetic nervous system are needed.

6-hydroxydopamine (6-OHDA) is a catecholaminergic neurotoxin that causes cell death by entering the cells via monoamine transporters, increasing the production of reactive oxygen species by rapid auto-oxidation and disrupting energy metabolism [21]. Systemic dosing of 6-OHDA induces peripheral sympathetic loss and has been used to model cardiac denervation in rodents [22], [23], [24], [25], dogs [26], [27] and monkeys [28], [29]. Few of these studies have evaluated cardiac sympathetic innervation with *in vivo* imaging (reviewed in [13]). None of them have confirmed imaging results using postmortem immunohistochemical analyses.

We have recently developed a nonhuman primate model of cardiac sympathetic neurodegeneration and dysautonomia by systemic dosing of 6-OHDA [29]. We chose this species because monkeys are highly regarded and widely used for modeling classical PD motor symptoms. They are a valuable resource for preclinical testing of novel anti-parkinsonian therapies [30] making them an ideal platform for future evaluation of disease-modifying strategies that could affect motor and nonmotor symptoms. Our *in vivo* findings at 3 months post-neurotoxin included loss of cardiac sympathetic innervation measured by reduced uptake of [^11^C]meta-hydroxyephedrine (MHED; a catecholamine analogue) using PET, and reduced circulating catecholamines. Here we report the postmortem characterization of the model. Our analysis, in the same 6-OHDA-treated monkeys, confirms that 3 months after administration of systemic 6-OHDA, monkeys have cardiac sympathetic neurodegeneration and dysfunctional catecholamine production in chromaffin cells of the adrenal medulla.

## Material and Method

### Ethics statement

The present study was performed in strict accordance with the recommendations in the NIH Guide for the Care and Use of Laboratory Animals (1996) in an AAALAC accredited facility (Wisconsin National Primate Research Center, University of Wisconsin-Madison). Experimental procedures were approved by the Institutional Animal Care and Use Committee (IACUC) of the University at the Wisconsin-Madison (permit number: G00538). All efforts were made to minimize the number of animals used and to ameliorate any distress. The control monkey tissues were obtained from the WNPRC tissue bank. These monkeys were originally assigned to the University of Wisconsin-Madison IACUC experimental protocols G00101, G00591, G00423. Positive controls for immunohistochemical stainings included brain tissue from transgenic mice overexpressing α-synuclein (University of Cincinnati IACUC, permit number: 09-12-30-01) and brain tissue from a monkey fetus day 38 (University of Wisconsin-Madison IACUC, permit number: G00452).

### Subjects

Cardiac and adrenal medulla sections from 10 rhesus monkeys (*Macaca mulatta*, 4 male, 5–17 yrs old) were utilized in this study. The animals from which the tissue was obtained were individually housed in Group 3 or Group 4 enclosures (cage floor area 4.3 ft.2 or 6.0 ft.2 per animal, height 30 or 32 in.) in accordance with the Animal Welfare Act and its regulations and the Guide for the Care and Use of Laboratory Animals (7th edition, 1996) with a 12-hour light/dark cycle. Throughout the study, the animals were monitored twice daily by an animal research or veterinary technician for evidence of disease or injury (e.g., inappetance, dehydration, diarrhea, lethargy, trauma, etc.), and body weight was monitored to ensure animals remained in properly sized cages. Animals were fed commercial nonhuman primate chow (2050 Teklad Global 20% Protein Primate Diet, Harlan Laboratories, Madison, WI) twice daily, supplemented with fruits or vegetables and a variety of forage items and received *ad libitum* water. Nonhuman primate chow soaked in a protein-enriched drink (Ensure©, Abbott Laboratories, Abbott Park, IL) was offered to stimulate appetite as needed.

The normal cardiac and adrenal medulla sections (n = 5; 10.76±4.29 yrs old; 7.75±4.55 kg; 2 male) were obtained from the tissue bank at the Wisconsin National Primate Center; the donors were selected by matching them to the baseline condition of the 6-OHDA-treated monkeys (normal adult rhesus monkeys, without previous history of cardiac dysfunction). The 6-OHDA-treated cardiac and adrenal medulla sections (n = 5; 7.0±1.57 yrs old; 7.35±1.65 kg; 2 male) were from a previously published study [29]. Dosing of 6-OHDA to these monkeys was done in sterile surgical conditions under isofluorane anesthesia, as a sequence of 7–9 intravenous injections totaling a final dose of 50 mg/kg. The animals were necropsied 3 months post-6-OHDA. The number of subjects per group was defined by our previous cardiac study [29] in which n = 5 found *in vivo* a statistically significant loss of cardiac denervation. Tissues from both groups were processed in parallel for each staining to minimize bias.

### Necropsy and tissue preparation

All animals were anesthetized with sodium pentobarbital (25 mg/kg, iv) and perfused through the ascending aorta or left atrium with heparinized phosphate-buffered saline, followed by 4% paraformaldehyde. Hearts were post-fixated in 4% paraformaldehyde for 24–48 hours and further preserved with 70% ethanol at 4°C. Hearts were carefully placed in a calibrated polymerized methyl methacrylate slice apparatus, laying on the inferior wall. From the base to the apex, the hearts were cut into 4 mm sections in a transverse plane to match the orientation of the left ventricle displayed in the MHED PET polar maps. A 1 mm punch was placed in the anteroseptal myocardium to mark orientation and sections were blocked in paraffin. Adrenals were post-fixated in 10% neutral buffered formalin, trimmed and blocked in paraffin. All blocked tissue was cut on a standard rotary microtome in 5µm section thickness and mounted on positively charged slides.

### General immunohistochemistry

Cardiac sections were deparaffinized and treated for heat antigen retrieval in a microwave for 6 minutes at 100% power followed by 6 minutes at 80% power. The sections were then washed and endogenous peroxidase activity blocked by incubation of 30% H_2_O_2_ and methanol. Nonspecific binding sites were blocked with a 10% serum and 0.5% BSA solution for 30 minutes at room temperature and incubated overnight with a primary antibody diluted in blocking buffer plus 0.1% Triton-X. The antibodies used included tyrosine hydroxylase (TH; 1∶400; Immunostar, Hudson, WI), protein gene product 9.5 (PGP9.5; 1∶200; Abcam, Cambridge, MA), human leukocyte antigen (HLA-DR; 1∶400; MP Biomedical, Santa Ana, CA), and nitrotyrosine (1∶300; Millipore, Temecula, CA). The sections were than incubated in appropriate biotinylated secondary antibody (1∶200), followed by avidin-biotin-peroxidase complex (ABC Standard, Vector Laboratories, Burlingame, CA), and visualized with a commercial 3,3′-diaminobenzidine kit (Vector Laboratories, Burlingame, CA). HLA-DR and nitrotyrosine staining was enhanced by the addition of ammonium nickel sulfate. Cardiac sections were counterstained with hematoxylin, dehydrated and coverslipped (Cytoseal mounting media, Thermo Scientific, Waltham, MA).

Adrenal tissue sections were immunostained for TH (1∶6,000), aromatic L-amino acid decarboxylase (AADC; 1∶500; Millipore, Billerica, MA), PGP9.5 (1∶200) and the mitochondrial import receptor unit TOMM20 (1∶25; Abcam, Cambridge, MA) following the procedure described above. PGP9.5 immunostaining was enhanced with ammonium nickel sulfate. TOMM20 immunostaining was counterstained with hematoxylin. Adrenal medulla sections were dehydrated, and coverslipped.

Negative controls were performed in parallel by omitting the primary antibodies in the immunostaining procedures. Cardiac and adrenals tissues were also stained for hematoxylin and eosin (H&E) for general anatomical evaluations.

### α-synuclein immunohistochemistry

Cardiac and adrenal tissues were also immunostained for three types of α-synuclein: soluble, phosphorylated at serine 129 and insoluble or proteinase K resistant. Brain tissue from Thy1-α-synuclein transgenic mice, also blocked in paraffin and cut at 5µm, was used as a positive control.

To assess soluble α-synuclein expression in the heart, the tissue was immunostained with similar steps as mentioned above, plus a repeated sequence with the first primary antibody incubation at a concentration of 1∶100 and the second primary incubation at 1∶50 (Invitrogen, Carlsbad, CA). The serum-blocking step was also modified and increased to 1 hour. Immunostaining of proteinase K resistant α-synuclein was conducted using the general immunohistochemistry protocol, with the addition of proteinase K incubation (1∶4,000; Life Technologies, Grand Island, NY) for 6 min before the endogenous peroxidase block and primary antibody incubation for two nights at 1∶50. Phosphorylated α-synuclein (1∶50; Abcam, Cambridge, MA) staining was performed with the antigen retrieval solution including 0.05% Tween-20 and overnight primary antibody incubation.

In the adrenal, immunostaining for soluble α-synuclein followed the general immunohistochemistry protocol with modifications of increased serum blocking to 1 hour and primary antibody incubation for two nights at 1∶50. Immunostaining for proteinase K resistant α-synuclein (1∶50) was incubated with proteinase K for 4 min and phosphorylated α-synuclein (1∶50) was performed similar to the heart tissue.

### Immunofluorescence

Immunofluorescence stainings were performed in cardiac tissue to identify catecholaminergic phenotype (by colocalization of TH-immunoreactivity (ir) and PGP9.5-ir) and sympathetic re-innervation (by TH-ir and growth associated protein 43 (GAP43)-ir) in cardiac nerve bundles. Slides were deparaffinized and antigen retrieval performed as described above. Tissue was blocked with 5% donkey serum and 2% BSA solution and incubated in primary antibodies GAP43 (1∶100; Millipore; Billerica, MA), PGP9.5 (1∶100), and TH (1∶200) for 2 days in 4°C. The sections were then incubated with alexafluor-conjugated secondary antibody (1∶1000) against the appropriate species and coverslipped with mounting media with DAPI (Vector Laboratories, Burlingame, CA). Brain tissue from a rhesus fetus at embryonic day 38 was used as a positive control for GAP43.

### Anatomical evaluation

Seven to eight representative H&E stained cardiac sections of each entire left ventricle and at least 1 section of adrenal gland per animal were blindly evaluated by a board certified veterinary pathologist (HAS). Cardiac sections throughout the myocardium, endocardium, epicardium and perivascular regions and adrenal sections throughout all cortical regions and the medulla were evaluated for histological changes such as presence and severity of inflammation, atrophy, or mineral deposits.

### Quantification of cardiac sympathetic innervation

Sympathetic innervation of the left ventricular myocardium, identified as either nerve bundles (collection of axons) or individual fibers, was evaluated using cardiac tissue immunostained for TH and PGP9.5. Assessment of epicardial bundles was not performed because immunohistochemical process resulted in inconsistent presence of the epicardium across animals. Quantification was performed in 4 matching slides per animal. Analysis of cardiac nerve bundles and fibers was reported as global innervation, further analyzed by left ventricle walls (anterior, inferior, lateral and septal) and levels (from apex to the base) and compared to previously obtained *in vivo* [^11^C]MHED PET polar maps [29]. In addition, cardiac bundle analysis considered differences between layers of the left ventricle (subepicardium and subendocardium).

TH-ir and PGP9.5-ir were analyzed in myocardial nerve bundles with areas >30 µm^2^ using a Zeiss Axioimager M2 equipped with a Qimaging camera and NIH ImageJ software. Images of left ventricle nerve bundles were captured (up to 6 per wall, 3 in the subepicardium and 3 in the subendocardium) and DAB color separated from hematoxylin counterstain with ImageJ Colour Deconvolution filter. ImageJ was calibrated using a step tablet, grey scale values were converted to optical density (OD) units using the Rodbard function, bundles were outlined and mean OD and area above threshold (AAT) measured using a threshold of 0.7 (TH) and 0.78 (PGP9.5) OD units.

TH-ir and PGP9.5-ir fibers were quantified using a state-of-the-art Zeiss Axioimager M2 microscope and the area fraction fractionator (AFF) probe function in StereoInvestigator v10.0 (MicroBrightField, Williston, VA). This software allows for automated selection of counting frames and estimates the fiber area of investigator-identified structures within a region of interest (ROI). ROIs were outlined under a low magnification (2.5x) for each wall of the left ventricle, including the anterior, inferior, lateral and septal walls. ROIs were defined as 8 mm wide and traced by drawing perpendicular lines from the left ventricular lumen to the epicardium joined by lines following the epicardial and endocardial borders (mean ROI = 43,932 mm^2^). Using a 63x oil immersion objective, the AFF randomly placed the 13.5 mm^2^ counting frame within the ROI and continued throughout the tissue moving with x and y sampling grids of 1 mm. In each counting frame a 2 µm grid was placed and an investigator highlighted the area of the grid with immunostained nerve fibers. TH-ir or PGP9.5-ir in nerve bundles >30 µm^2^ were excluded during fiber area density procedure. An estimated area per region was calculated by the AFF probe, and reported as area density of TH-ir or PGP9.5-ir in fibers (µm^2^) over the total area of ROI (mm^2^). The area density values for each ROI within an individual slide were summed and the sum per slide used to evaluate differences in sympathetic fiber innervation at different levels (apical to basal) of the heart. Global and wall analyses were calculated by adding the estimated areas across 4 slides.

### GAP43 evaluation

Presence of GAP43 positive immunofluorescense was evaluated in one representative cardiac slide per animal that was matched for level. Intensity and extension of the staining was quantified in 3 cardiac nerve bundles per wall of the left ventricle (total 12 nerve bundles) using a semi-quantitative scale. The scoring system was defined as 0 = absent, 1 = mild (<25% peppered throughout bundle), 2 = moderate (25–75% peppered throughout bundle), and 3 = severe (>75% peppered throughout bundle). Scores per slide were averaged and compared across groups.

### Nitrotyrosine quantification

Nitrotyrosine-ir was evaluated in 4 matching cardiac slides per animal spanning the levels of the heart. The left ventricle walls were outlined and a nerve bundle in close proximity to a cardiac vessel was randomly selected at 10x in each wall of the left ventricle. Blood vessels, nerve bundles, and cardiomyocytes were blindly evaluated at 40x, and process repeated in up to 4 sites per wall. If 4 nerve bundles could not be identified at 10x, a random large vessel with diameter >0.8 µm was selected and area magnified to 40x to detect nerve bundles that were not easily observed at 10x.

As blood vessels, nerve bundles, and cardiomyocytes presented distinct localization of nitrotyrosine, they were rated separately using similar scoring values (0–4) but specific semi-quantitative rating scales. The scale for cardiomyocytes was defined as: 0 = absence of nitrotyrosine staining, 1 = mild, 2 = stained border that lightens in the center, 3 = light stain throughout cell and 4 = dark stain throughout the cell. For vasculature: 0 = absent, 1 = 25–50% peppered stain around perimeter of vessel, 2 = <50% light stain around perimeter of vessel, 3 = >50% light stain around perimeter of vessel, and 4 = >50% dark stain around perimeter of vessel. For nerve bundles: 0 = absent, 1 = <50% stain, 2 = 50–75% stain, 3 = 75–100% light stain, 4 = 100% stain. Individual scores were summed across wall for regional analysis and all regions summed for global results.

### HLA-DR quantification

Presence of HLA-DR positive macrophage-like cells in the left ventricle was quantified in 4 matching cardiac slides per animal. The walls of the left ventricle were individually surveyed at 10x and areas of HLA-DR expression magnified at 40x. The number of HLA-DR fields of view at 40x was totaled for each wall and summed across all walls and slides for global analysis.

### α-synuclein quantification

Each α-synuclein immunostaining (soluble, phosphorylated and insoluble) was evaluated in two matched cardiac slides per subject. Positive nerve bundles were identified at 10x and digital images obtained at 63x using a Zeiss Axioimager M2 microscope. Presence and intensity of immunoreactivity was analyzed in 3 nerve bundles for each of the 4 walls of the left ventricle using a semi-quantitative scale. The scale was defined as 0 = absent, 1 = mild intensity, 2 = moderate intensity, and 3 = severe intensity. Individual animal scores were averaged across slides (to obtain a total score) and across walls (regional score) and then averaged across groups.

### Quantification of adrenal medulla immunostainings

Expression of TH, AADC, PGP9.5, TOMM20 and α-synuclein were quantified in one adrenal section per staining, per animal. For TH and AADC, images were captured at low magnification (2x) using a Nikon E800 microscope equipped with a SPOT camera, while images of PGP9.5, TOMM20 and α-synuclein were taken at 2.5x on a Zeiss Axioimager M2 equipped with a Qimaging camera. Using ImageJ NIH software, 3 ROIs were drawn throughout the adrenal medulla avoiding any vasculature >2.5 µm^2^. AAT and OD quantifications were collected similarly as the cardiac bundle evaluations, but instead with a threshold of 0.2 OD units. TOMM20 images used ImageJ Colour Deconvolution filter to separate hematoxylin and DAB staining. AAT and OD values were averaged within an individual animal and then across groups. Soluble and insoluble α-synuclein-ir was calculated using particle count function with object size 0–200 pixels^2^. The total particle number for one representative ROI was recorded and compared across groups. Phosphorylated α-synuclein was evaluated with a semi-quantitative scale similar to cardiac α-synuclein rating.

### Statistical analysis

Data collection and analysis were performed by investigators blind to the treatment groups. All statistical analysis was performed using GraphPad Prism (version 5.0b, GraphPad Software) or with SPSS (version 18, Chicago, IL). A *P* value <0.05 was accepted as significant. Group averages for global and regional cardiac and adrenal evaluations were analyzed with Independent sample t-test. Regional differences between walls within the 6-OHDA or control group alone were evaluated using a one-way ANOVA with *post hoc* analysis using Bonferroni multiple comparisons. Deficits were expressed as percentages and calculated by subtracting the 6-OHDA value from the average control measurement and dividing by the average control. Pearson's correlations were performed to determine the association between cardiac histological measures and *in vivo* MHED PET distribution volumes obtained from a previously published study performed in the same 6-OHDA-treated animals [29]. The MHED PET baseline values (before neurotoxin exposure) of the previous report were averaged within each wall of the left ventricle, and used as control values for comparison.

## Results

### H&E stained cardiac and adrenal tissue showed minimal changes

**Figure 1:**
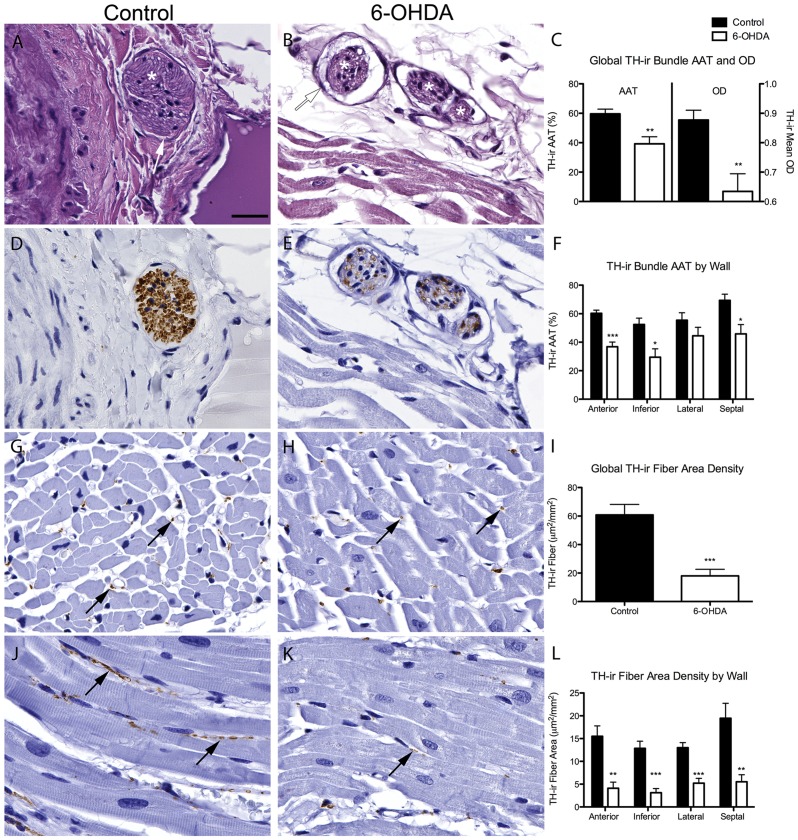
6-OHDA-treated monkeys have reduced TH-ir in cardiac nerve bundles and fibers. (**A,B**) Microphotographs of H&E stained nerve bundles identified as a collection of nerve fibers (white asterisk) and intact epineurium indicated by white arrows. (**C**) Global TH-ir in cardiac nerve bundle was reduced for both AAT (t(8) = 3.508, *P* = 0.008) and OD quantification (t(8) = 3.403, *P* = 0.009) in 6-OHDA compared to control animals. (**D,E**) Microphotographs of TH-ir in matching nerve bundles. (**F**) Regional wall quantification showed reduced AAT of TH-ir in anterior (t(8) = 5.913, *P*<0.0001), inferior (t(8) = 3.096, *P* = 0.015), and septal (t(8) = 3.009, *P* = 0.017) walls in 6-OHDA-treated monkeys compared to controls. In the control group there were also significant differences in OD quantification of TH (F(3,16) = 5.213, *P* = 0.011) with more TH-ir in the septal wall compared to the lateral (*P* = 0.043) and inferior walls (*P* = 0.12) (*post hoc* Bonferroni multiple comparisons analysis; not shown). (**G,H,J,K**) Microphotographs of cardiac TH positive fibers in either transverse (**G,H**) or longitudinal orientation (**J,K**) in control and 6-OHDA monkeys; black arrows indicate TH-ir fibers. (**I,L**) TH-ir fiber area density was significantly reduced in 6-OHDA compared to control animals for global (**I**) analysis (t(8) = 4.934, *P* = 0.001) and between all walls of the left ventricle (**L**) including the anterior (t(8) = 4.324, *P* = 0.003), inferior (t(8) = 5.37, *P* = 0.001), lateral (t(8) = 5.081,*P* = 0.001) and septal walls (t(8) = 3.866, *P* = 0.005). Scale bar  = 25 µm. *P<0.05, **P<0.01, ***P<0.001. AAT, area above threshold; OD, optical density; TH, tyrosine hydroxylase.

General pathological evaluation of H&E stained cardiac sections showed in all animals normal ventricular myocytes displaying centrally placed nuclei, intercalated discs and cross-striations. Cardiac nerve bundles presented normal morphology with intact nerve fibers encased by epineurium, defined as a collagenous sheath composed of flattened cells with elongated nuclei. Separation of myofibers and connective tissue as well as separation or shrinkage of nerve bundles were observed in both groups (Figure 1A,B) and were consistent with artifacts caused by sectioning and processing. Variable minimal to mild amounts of lymphocytic infiltration in the myocardium and surrounding coronary vessels were noted in 6-OHDA-treated animals. Differences between individuals were observed between regions (from the cardiac apex to the base), number of areas affected and proportions of perivascular vs. myocardial locations. One 6-OHDA-treated animal had saponification of pericardial adipose tissue with lymphocytes, plasma cells and neutrophils. One control animal (17 yrs old) had moderate degenerative and fibrosing cardiomyopathy characterized by moderate multifocal myofiber degeneration and loss with increased interstitial fibrosis, a typical findings in older adult animals of the WNPRC colony.

**Figure 2:**
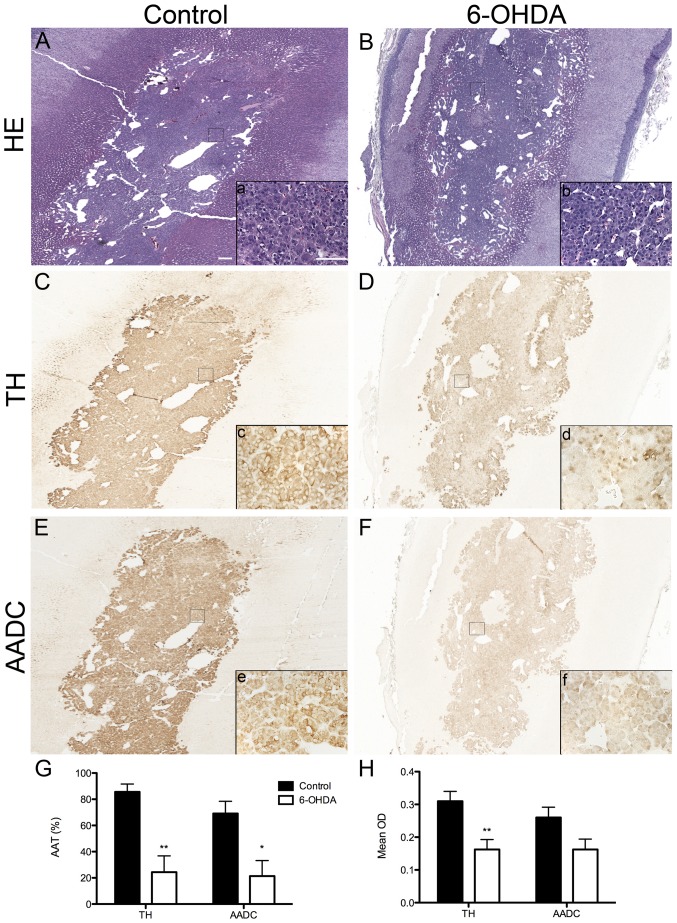
6-OHDA-treated monkeys have reduced TH-ir and AADC-ir in the adrenal medulla. (**A-F**) Microphotographs of adrenal medulla sections stained for H&E (**A,B**), TH (**C,D**) and AADC (**E,F**) from control and 6-OHDA monkeys. Squares in A-F corresponds to respective insets a-f showing a higher magnification image of the selected area. (**G**) OD (TH: t(8) = 3.455, *P* = 0.009; AADC: t(8) = 2.192, *P* = 0.06) and (**H**) AAT (TH: t(5.791) = 4.437, *P* = 0.005; AADC: t(8) = 3.180, *P* = 0.013) quantifications of immunostainings showed significant reductions in 6-OHDA monkeys. Scale bar  = 50 µm; inset scale bar  = 50 µm. *P<0.05, **P<0.01. AADC, aromatic l-amino acid; AAT, area above threshold; OD, optical density; TH, tyrosine hydroxylase.

Evaluation of H&E stained adrenal medulla sections in all animals showed columnar shaped chromaffin cells with normal granular cytoplasm clustered around the medullary network of capillaries. Focal mineral deposits were observed in 50% of the animals (approximately equal numbers of control and experimental), a common histological finding in adult rhesus monkeys [31], [32] and baboons [33]. A single incidental focus of fibrosis was detected in the medulla of a 6-OHDA-treated animal; no other significant lesions were observed in the adrenal medulla (Figure 2A,B).

### 6-OHDA-treated animals have reduced cardiac TH-ir nerve bundles and fibers

TH expression, used as a marker of catecholaminergic innervation, was found in axons of cardiac nerve bundles and presented as dots (transverse orientation) or wavy lines (tangential orientation) immunostaining. Occasionally TH immunostaining in nerve bundles was intense, did not stay in the defined boundaries of individual processes, and was diffusely distributed inside the epineurium, covering axons and the area surrounding the axons.

TH-ir was reduced in cardiac nerve bundles of 6-OHDA-treated monkeys compared to controls (Figure 1D,E). Quantitative analysis of TH-ir confirmed a significantly reduced global AAT (34%) and OD (28%) in nerve bundles of 6-OHDA-treated animals (Figure 1C). Across left ventricle walls, AAT quantification of TH-ir was significantly decreased in the anterior, inferior, and septal walls in 6-OHDA-treated animals compared to controls with less TH deficit in the lateral wall (Figure 1F). Patterns of TH expression in nerve bundles across walls and cardiac levels were further investigated by analyzing each group separately. Evaluation of the left ventricle walls in the control group showed significant differences in OD TH-ir with the greatest TH expression in the septal wall. In the 6-OHDA group, no significant differences between walls were found, suggesting a more homogenous innervation. Within both groups, TH expression in nerve bundles across cardiac levels (apex to the base) and between the subepicardium and the subendocardium layers did not show statistically significant differences.

TH immunostaining was also detected in cardiac sympathetic nerve fibers throughout the myocardium and resembled single punctate structures (transverse orientation; Figure 1G,H) or elongated dots strung together but not necessarily continuous (longitudinal orientation; Figure 1J,K). Qualitatively, TH-ir fibers seemed to be reduced in 6-OHDA-treated monkeys compared to controls. Quantification of TH-ir fibers showed significantly reduced global area density (70% average deficit) in 6-OHDA animals compared to controls (Figure 1I). Across left ventricle walls, there were significant losses in TH-ir fiber area density in anterior (73%), inferior (76%), and septal (72%) compared to controls, with less loss in the lateral wall (60%), similar to TH expression in nerve bundles (Figure 1L). Quantification of TH positive fibers showed no significant differences across apex to base levels. In the control group there was a significant correlation between age and TH-ir area fiber density (r2 = 0.7750, P = 0.0488). Interestingly, the older animals had greater TH-ir area fiber density, suggesting that age did not adversely effect sympathetic innervation, probably because they were all adult and not postmenopausal age rhesus monkeys (e.g.: >25 yrs old).

**Figure 3:**
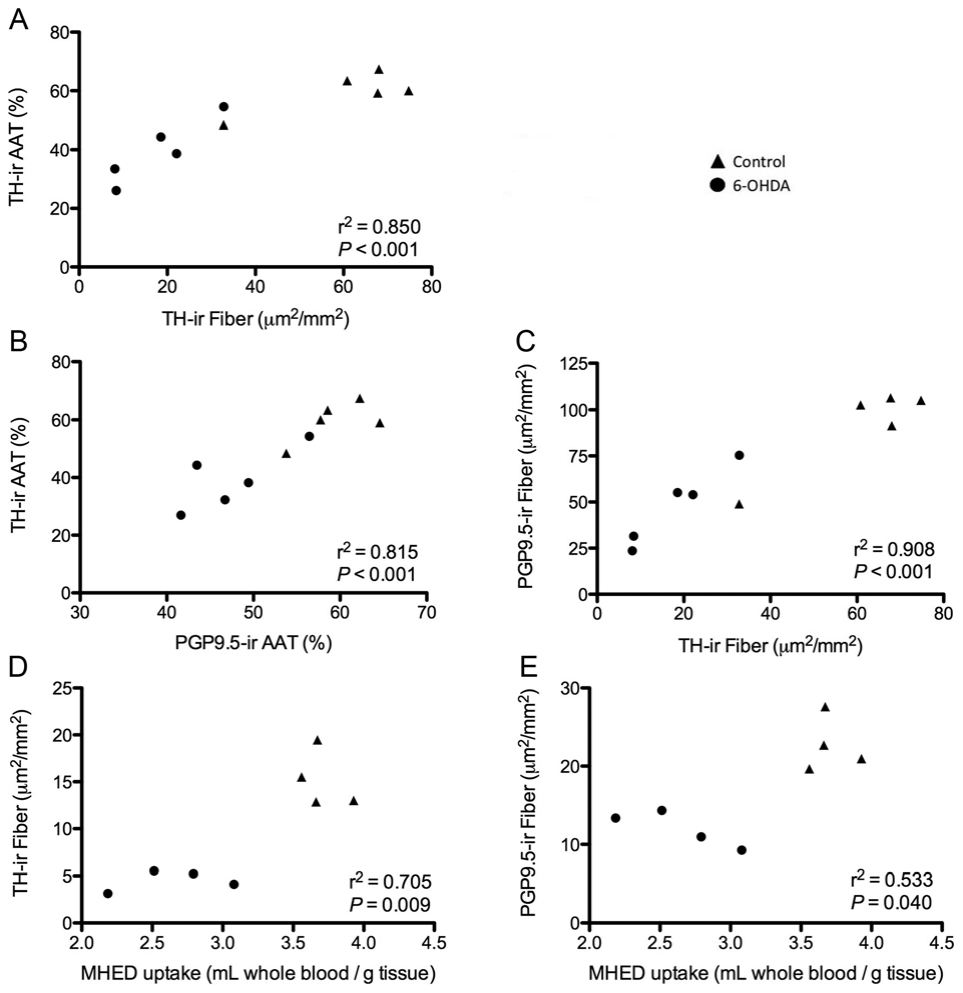
Measures of cardiac TH and PGP9.5 expression correlated with *in vivo* MHED uptake. Nerve bundle AAT quantification of TH-ir correlated with TH-ir fibers (**A**) and AAT of PGP9.5-ir nerve bundles (**B**). Area density quantification of TH-ir fibers highly correlated with PGP9.5-ir fibers (**C**). *In vivo* MHED uptake correlated with TH-ir (**D**) and PGP9.5-ir fiber area density (**E**). Despite the significant interaction between MHED and TH-ir fibers, MHED uptake did not correlate with AAT quantification of TH-ir nerve bundles (r^2^ = 0.446, *P* = 0.070) (not shown). AAT, area above threshold; MHED, meta-hydroxyephedrine; PGP9.5, protein gene product 9.5; TH, tyrosine hydroxylase.

Correlation analysis between area density of TH-ir in fibers and AAT of TH-ir nerve bundles showed a positive association between both measures (Figure 3A). Comparisons between regional wall measures of TH-ir with previously reported *in vivo* MHED data (Figure 3D) showed a significant correlation with averaged TH positive fiber area density by wall and a trend with AAT of TH-ir in nerve bundles.

### Decreased PGP9.5-ir cardiac nerve bundles and fibers confirms neurodegeneration

**Figure 4:**
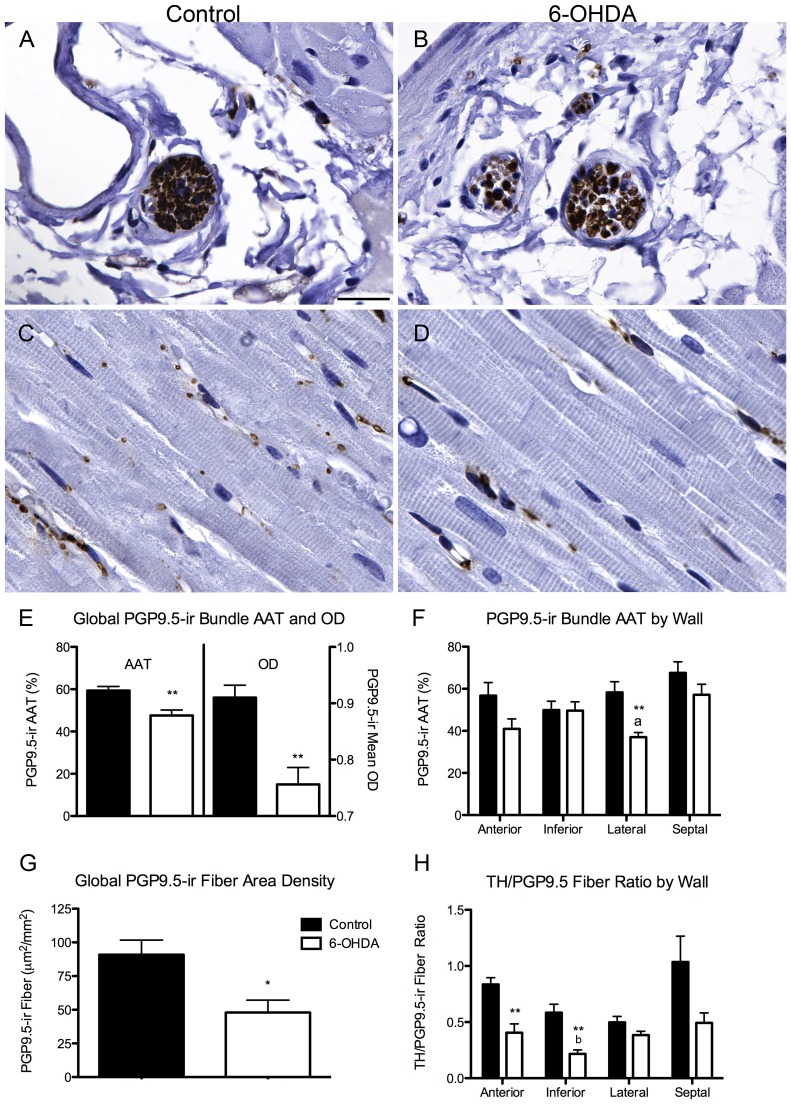
6-OHDA-treated monkeys have decreased PGP9.5-ir in cardiac nerve bundles and fibers. (**A–D**) Microphotographs of PGP9.5-positive nerve bundles (**A,B**) and fibers (**C,D**) in control and 6-OHDA-treated animals. (**E**) Global PGP9.5-ir cardiac nerve bundle was reduced for both AAT (t(8) = 3.7, *P* = 0.006) and OD quantification (t(8) = 4.21, *P* = 0.003) in 6-OHDA compared to control animals. (**F**) PGP9.5-ir nerve bundle AAT was also decreased between groups in the lateral wall of the left ventricle. Within the 6-OHDA group, a significant effect of wall (ANOVA: F(3,16) = 4.632, *P* = 0.016) with the greatest reduction in the lateral compared to the septal wall (^a^ *P* = 0.022) was found. (**G**) Quantification of PGP9.5-ir fiber area density was significantly reduced in 6-OHDA-treated monkeys compared to controls (t(8) = 3.026, *P* = 0.016). (**H**) TH to PGP9.5 positive fibers ratio was significantly decreased in the anterior (t(8) = 4.335, *P* = 0.002) and inferior walls (t(8) = 4.43, *P* = 0.002) in 6-OHDA animals compared to controls. Within the 6-OHDA group there was a significant reduction in ratio between walls (ANOVA; F(3,16) = 3.299, *P* = 0.047) with significant loss in the inferior compared to septal wall (^b^ *P* = 0.044). Scale bar  = 25 µm. *P<0.05, **P<0.01, ^a^ *P* = 0.02, ^b^ *P* = 0.044. AAT, area above threshold; OD, optical density; PGP9.5, protein gene product 9.5; TH, tyrosine hydroxylase.

To confirm cardiac nerve loss, pan-neuronal marker PGP9.5-ir was evaluated in nerve bundles and fibers. PGP9.5-ir cardiac nerve bundles presented morphology similar to TH immunostaining with expression found in axons either in a transverse or tangential plane that were surrounded by an epineurium lining (Figure 4A,B). Comparison between 6-OHDA-treated animals and controls showed a significant decrease in global AAT (20%) and OD (17%) quantification of PGP9.5 positive nerve bundles, corroborating the loss in TH expression (Figure 4E). Analysis between groups and across walls of the left ventricle showed significantly lower AAT of PGP9.5-ir in nerve bundles in the lateral wall (37%). Analysis within the control group did not show differences between walls; within the 6-OHDA group, PGP9.5-ir in nerve bundles had the greatest reduction in the lateral compared to the septal wall (Figure 4F).

Cardiac nerves fibers positive for PGP9.5 seemed to be diminished in 6-OHDA-treated animals compared to controls (Figure 4C,D). Quantitative analysis of PGP9.5 cardiac fibers demonstrated a significant area density reduction (47%) in 6-OHDA monkeys compared to controls (Figure 4G). Regional quantification demonstrated significant reductions of PGP9.5-ir fiber area density in the anterior (t(8) = 2.402, *P* = 0.043), inferior (t(8) = 2.563, *P* = 0.034) and septal wall (t(8) = 2.396, *P* = 0.043) of 6-OHDA-treated monkeys compared to controls.

**Figure 5:**
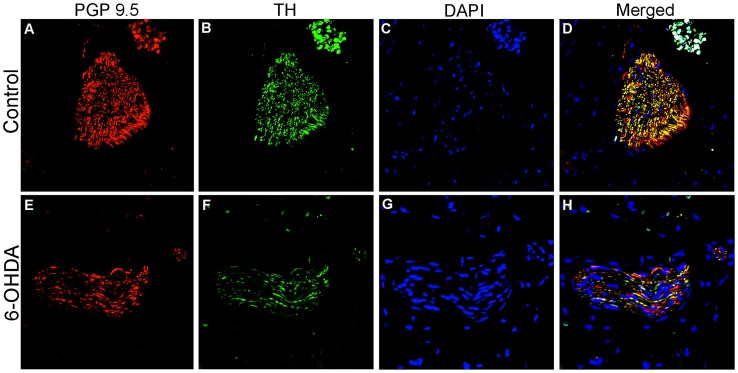
PGP9.5 and TH immunofluorescense demonstrated colabeling and decreased expression in cardiac nerve bundles of 6-OHDA monkeys. Microphotographs of immunofluorescence staining of PGP9.5 (red; **A,E**), TH (green; **B,F**) and counterstained with DAPI (blue nuclei; **C,G**) in controls (A–D) and 6-OHDA-treated animals (E–H). Colocalization of PGP9.5 and TH expression is identified as yellow. PGP9.5, protein gene product 9.5; TH, tyrosine hydroxylase.

Significant correlations were found between AAT quantification of PGP9.5-ir and TH-ir in nerve bundles (Figure 3B), as well as between fiber area density (Figure 3C), suggesting similar loss. Immunofluorescence colabeling of PGP9.5 and TH expression showed a reduction in both markers in cardiac nerve bundles of 6-OHDA animals (Figure 5) and confirmed the loss of sympathetic innervation to the left ventricle.

The ratio of TH-ir/PGP9.5-ir fiber area density across all 4 walls of the left ventricle was lowest in the inferior wall (63%) in 6-OHDA animals similar to the TH-ir fiber area density (Figure 4H). Evaluation of 6-OHDA animals alone revealed a significant reduction in ratios between walls with a significant loss in the inferior compared to septal wall. The regional decline in TH/PGP9.5 fiber ratio matched the *in vivo* reduced capacity of the inferior wall to take up MHED relative to blood. Averaged area density of PGP9.5-ir fibers by wall showed a significant correlation with regional MHED uptake (Figure 3E).

### Increased GAP43-ir in 6-OHDA-treated animals reflects regenerating axons

**Figure 6:**
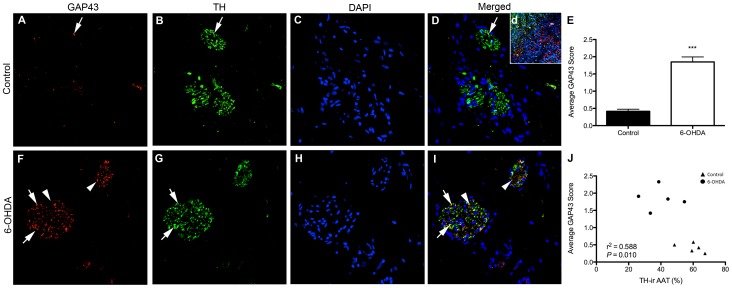
GAP43 immunofluorescence increased in 6-OHDA monkeys and had minimal colabeling with TH. Cardiac sections of control (**A–D**) and 6-OHDA-treated (**F–I**) animals were labeled for GAP43 (red) and TH (green) immunofluorescence and counterstained with DAPI (blue nuclei). Arrows indicate GAP43 expression in nerve bundles. Arrowheads indicate colocalization of GAP43-ir and TH-ir. Inset (**d**) shows a positive control for GAP43 labeling in caudorostral diencephalon of rhesus embryonic day 38, run in parallel to these stainings. (**E**) Averaged scores demonstrate an increase of GAP43-ir in 6-OHDA monkeys. (t(8) = 9.073, *P*<0.001). (**J**) Global GAP43 scores negatively correlate with AAT of TH-ir in cardiac nerve bundles. ***P<0.001. AAT, area above threshold; GAP43, growth associated protein 43; TH, tyrosine hydroxylase.

Cardiac GAP43 immunofluorescence was used to identify potential recovery of innervation following 6-OHDA. GAP43-ir was preferentially observed in axons inside cardiac nerve bundles of 6-OHDA-treated monkeys (Figure 6). Quantification confirmed significantly higher average GAP43-ir score in 6-OHDA animals compared to controls (Figure 6E), suggesting axonal regrowth 3 months post-neurotoxin exposure. No significant differences were found among left ventricle walls. Global GAP43-ir scores negatively correlated with AAT quantification of TH-ir in cardiac nerve bundles (Figure 6J), suggesting animals treated with systemic 6-OHDA have less sympathetic innervation and greater expression of GAP43 in cardiac bundles.

### Nitrotyrosine and HLA-DR expression showed minimal differences between groups

**Figure 7:**
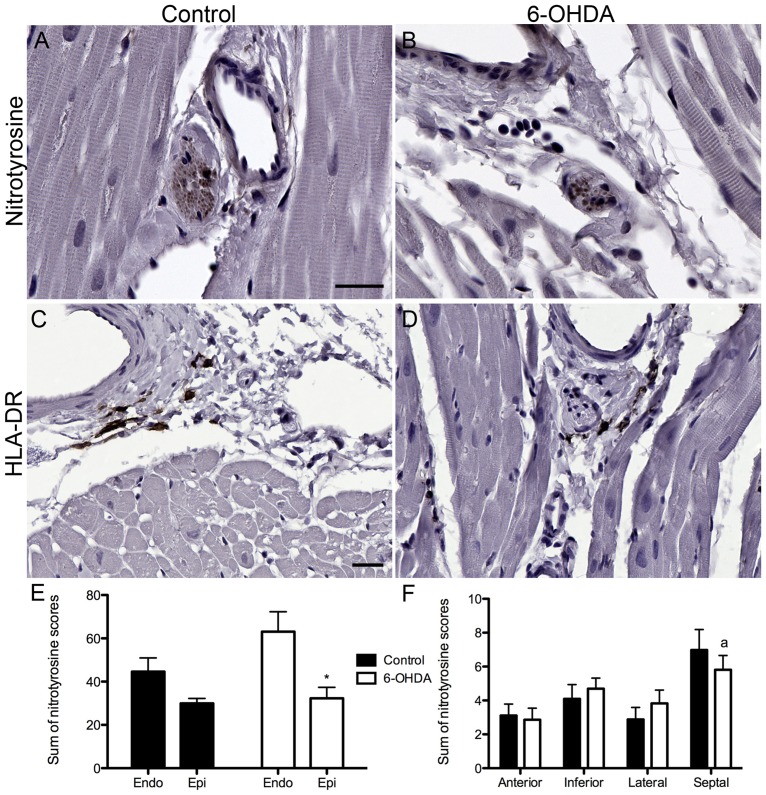
Similar nitrotyrosine-ir and HLA-DR-ir were observed across groups. (**A–D**) Microphotographs of a region of the left ventricle containing a nerve bundle, blood vessel and cardiomyocytes immunostained for nitrotyrosine (**A,B**) and human leukocyte antigen (HLA-DR) (**C,D**) in control (**A,C**) and 6-OHDA-treated monkeys (**B,D**). (**E**) The sum of nitrotyrosine scores in 6-OHDA-treated animals was significantly lower in the subepicardial compared to the subendocardial layer (t(8) = 2.921, *P* = 0.019), while no difference were found between layers in the control group. (**F**) Summed nitrotyrosine scores in nerve bundles by wall were not different between groups, but within 6-OHDA-treated animals a significant effect was found between walls (ANOVA: F(3,16) = 3.803, *P* = 0.031), with greater nitrotyrosine-ir scores in the septal than the anterior wall (^a^ *P* = 0.031). Scale bar  = 25 µm. *P = 0.019, ^a^ *P* = 0.031.

Nitrotyrosine expression (marker of oxidative stress) was detected in all animals' nerve bundles, blood vessels and cardiomyocytes at varying intensities. In nerve bundles nitrotyrosine-ir was observed as dots that resembled individual axons or, in the cases of more intense immunostaining, covering all areas inside the epineurium. In blood vessel walls and cardiomyocytes nitrotyrosine-ir was observed as diffuse intracytoplasmic shading (Figure 7A,B). Quantification of nitrotyrosine-ir with a semi-quantitative scale showed higher total sum score in 6-OHDA animals (28%) compared to controls, but did not reach statistical significant difference. Comparisons across walls or levels were also not significant, yet evaluation between subendocardial and subepicardium layers confirmed a significant difference in 6-OHDA-treated animals (Figure 7E). Comparison of nitrotyrosine-ir by cell type showed no significant differences between groups. Further analysis of nitrotyrosine positive nerve bundles by wall or level did not show significant differences between groups. When nitrotyrosine-ir scores for nerve bundles were compared between walls within each group, 6-OHDA monkeys showed significantly higher scores in the septal compared to the anterior wall.

Immunolabeling for HLA-DR was observed in animals from both groups, covering basophilic rich macrophage-like cells located perivascularly or between muscle cells (Figure 7C,D). Quantification of HLA-DR-ir was not significantly different between groups. It should be mentioned that two control animals had intense HLA-DR-ir; analysis of scores using Grubb's outlier test identified one of the subjects as an outlier and was removed from the analysis (n = 10, Z = 2.29, *P*<0.05). The effect of 6-OHDA found 3 months after intoxication did not seem to be greater than that normally found in normal adult monkeys, suggesting that the results would not be affected by the smaller n. HLA-DR-ir quantification did not correlate with measurements for innervation (PGP9.5 or TH), oxidative stress (nitrotyrosine), or neuronal recovery (GAP43).

### Soluble α-synuclein was moderately decreased in 6-OHDA-treated animals

**Figure 8:**
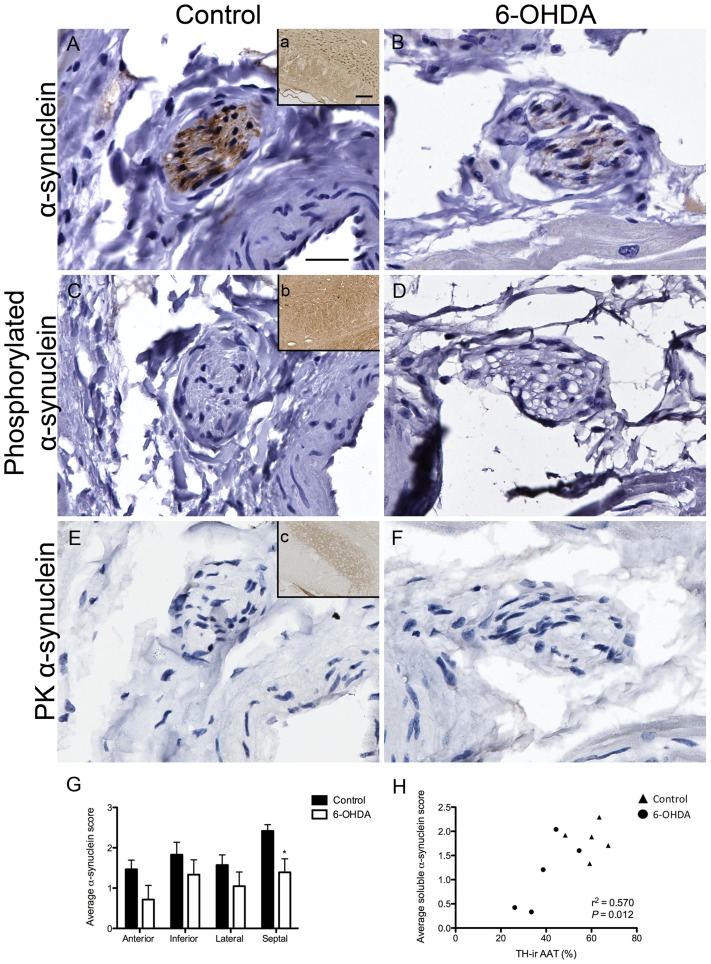
Cardiac α-synuclein was moderately affected by 6-OHDA. (**A–D**) Microphotographs of nerve bundles immunostained for soluble α-synuclein (**A,B**), phosphorylated α-synuclein (**C,D**) and proteinase K resistant α-synuclein (**E,F**) in control (**A,C,E**) and 6-OHDA-treated (**B,D,F**) monkeys. Insets (**a–c**) display immunostained nigral tissue from a Thy1-α-synuclein mouse processed in parallel for each specific antibody and used as a positive control to validate staining. (**G**) Regional reduction of soluble α-synuclein expression was found in the septal wall (t(8) = 2.787, *P* = 0.024) of 6-OHDA compared to control monkeys. Within each group, soluble α-synuclein scores did not show significant differences by wall in control animals (ANOVA; F(3,16) = 3.115, *P* = 0.056) and in 6-OHDA-treated animals (ANOVA: F(3,16) = 0.772, *P* = 0.526). (**H**) Average scores of soluble α-synuclein-ir in nerve bundles correlated with AAT TH-positive nerve bundles (r^2^ = 0.570, *P* = 0.012). Scale bar  = 25 µm, inset scale bar  = 100 µm. *P<0.05. AAT, area above threshold; TH, tyrosine hydroxylase.

Soluble α-synuclein expression was observed in all animals as diffuse shading inside the epineurium of cardiac nerve bundles, with areas of more intense staining often over hematoxylin stained nuclei (Figure 8A,B). Quantitative analysis found a trend in reduced global soluble α-synuclein-ir in cardiac nerve bundles and a significant regional reduction of soluble α-synuclein expression in the septal wall of 6-OHDA monkeys compared to controls (Figure 8G). Analysis within either group did not find significant differences by wall. Global soluble α-synuclein-ir scores correlated with AAT quantification of TH-ir in nerve bundles (Figure 8H), suggesting the reduction of soluble α-synuclein-ir is a consequence of sympathetic neurodegeneration.

Phosphorylated α-synuclein-ir was mostly absent in all animals (Figure 8C,D). In two control subjects and one 6-OHDA-treated animal phosphorylated α-synuclein-ir was observed as localized shading or more intense punctate structures inside nerve bundles, resembling cross-sections of individual axons. Due to its minimal expression phosphorylated α-synuclein-ir was not quantified. Proteinase K resistant α-synuclein-ir aggregates were not found in either control or 6-OHDA-treated animals (Figure 8E,F).

### Adrenal TH-ir and AADC-ir were reduced in 6-OHDA monkeys

TH-ir and AADC-ir in the adrenal medulla of all monkeys were observed as intracytoplasmic staining in chromaffin cells; both seem to be decreased in 6-OHDA-treated animals compared to controls (Figure 2C–F). Analysis of OD and AAT of TH-ir confirmed a significant reduction in 6-OHDA monkeys (Figure 2G), while AADC-ir quantification showed a trend in OD and a significant decrease in AAT in 6-OHDA monkeys compared to controls (Figure 2H).

**Figure 9:**
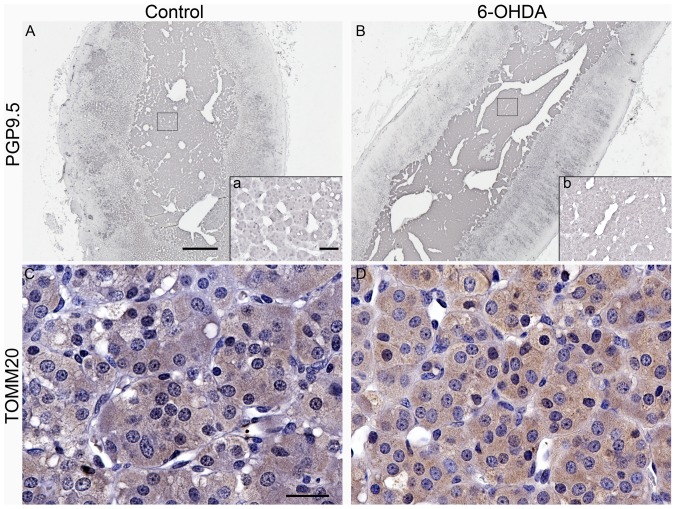
PGP9.5-ir and TOMM20-ir in the adrenal medulla were similar between groups. Microphotographs of adrenal medulla immunostained for PGP9.5 (**A,B**) and TOMM20 (**C,D**) from representative control (**A,C**) and 6-OHDA (**B,D**) monkeys. Squares in A and B corresponds to insets a and b, showing a higher magnification view of the selected area. Scale bar A,B = 500 µm, C,D = 25 µm; inset scale bar  = 50 µm.

PGP9.5-ir was detected in the cytoplasm of chromaffin cells, often as homogenous staining, and to a lesser extent scattered in cells of the zona reticularis and fasciculata of the adrenal cortex. TOMM20-ir was observed as diffuse intracytoplasmic staining in chromaffin cells (Figure 9C,D). In zona reticularis and fasciculata, TOMM20-ir was observed as intracytoplasmic punctate staining (not shown). No significant differences were found between groups for either AAT or OD quantification of PGP9.5-ir or TOMM20-ir in chromaffin cells.

**Figure 10:**
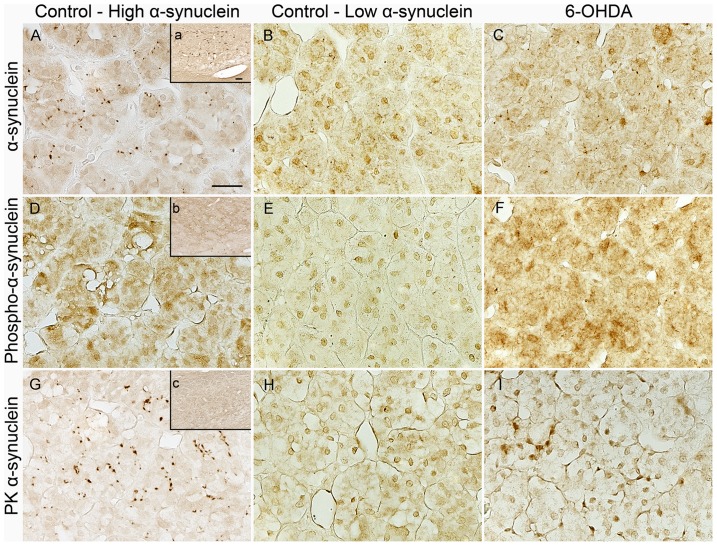
α-synuclein expression in the adrenal medulla was not affected by 6-OHDA treatment. Microphotographs of adrenal medulla immunostained for soluble α-synuclein (**A,B,C**), phosphorylated α-synuclein (**D,E,F**) and proteinase K resistant α-synuclein (**G,H,K**) in 6-OHDA-treated (**C,F,I**) and low or high α-synuclein expressing control monkeys. Insets display nigral tissue from a Thy1-α-synuclein mouse immunolabeled for each antibody in parallel and used as a positive control to validate processing. Scale bar  = 25 µm, inset scale bar  = 50 µm.

Intense soluble α-synuclein expression was observed in two control animals as dots sprinkled throughout the adrenal medulla, mostly following the inner perimeter of chromaffin cells; the rest of the animals had minimal to nearly absent immunostaining (Figure 10A–C). Due to the high variability of soluble α-synuclein expression in controls, quantitative analysis for particle counts found no significance difference between groups. Phoshorylated α-synuclein-ir was nearly absent in controls with low soluble α-synuclein-ir while some immunostaining was found in controls with high soluble α-synuclein-ir and 6-OHDA-treated animals as intracytoplasmic granules in chromaffin cells (Figure 10D–F). Quantitative analysis showed no difference in particle counts between groups. Sections stained for proteinase K resistant α-synuclein had variable cytoplasmic digestion in chromaffin cells and showed no aggregate immunostaining, with exception of one control animal with high soluble α-synuclein-ir that had areas of dotted insoluble protein interspersed between areas of digested cytoplasm (Figure 10G–I).

## Discussion

The present study demonstrates that systemic 6-OHDA dosing to rhesus monkeys induces cardiac sympathetic neurodegeneration and decreases catecholaminergic enzymes in the adrenal medulla. To the best of our knowledge, this is the first pathological report of a nonhuman primate model of peripheral sympathetic loss.

6-OHDA-treated monkeys, like many PD patients, had decreased sympathetic cardiac innervation, although the extent of loss seems to be less than the human cases. In our study quantification of cardiac TH expression throughout the myocardium revealed a 34% reduction in nerve bundles and 70% in fibers. In PD, TH positive epicardial bundles [14], [15], [34] and cardiac fibers extending into the myocardium [35] have been described as profoundly reduced (1–5% of controls) or absent compared to aged-matched controls. The reported human cases corresponded to patients with severe PD, suggesting that the extensive sympathetic loss may be related to the advanced stage and duration of disease at the time of evaluation, and that the monkey model may better represent earlier stages of the disease.

Another explanation for the differences in TH expression between PD and 6-OHDA monkeys may be related to the evaluated cardiac area and/or quantification methods. In PD patients, only epicardial bundles in the anterior wall have been measured by either calculating areas of immunoreactivity [14] or by counting individual axons in nerve bundles exceeding 1000 µm^2^ [34]. Quantification of nerve bundles or fiber area density throughout the myocardium is absent from PD literature. Although in normal cardiac tissue has been evaluated the total number of fields having TH-ir in nerves was calculated across a defined region in human hearts [36], while others have estimated the total axon length in the left ventricle of mice [37]. In contrast, in this study, we quantified TH-ir throughout the myocardium in both cardiac nerve bundles and fibers. We assessed the area of immunoreactivity as a function of the absolute nerve bundle area and estimated nerve area density using a novel method allowing systematic and unbiased counting. We conducted separate analyses of each of the four walls of the left ventricle to identify regional patterns of sympathetic innervation and did not evaluate epicardial nerve bundles because we found that this area was not consistently present in all animals after immunohistochemical processing. Further evaluation of PD tissues is necessary to understand the distribution of sympathetic loss deeper than the epicardial surface and throughout all walls of left ventricle.

Comparisons with other available reports on animal models of sympathetic denervation suggest that 6-OHDA is an effective catecholaminergic toxin. Reductions in cardiac norepinephrine levels were shown postmortem in mice 3 days post-toxin using a trihydroxyindole method [23] and up to 14 days post-toxin using paper chromatography [38], in rats 5 days post-toxin using HPLC [39], and in dogs 5 days post-toxin using a trihydroxyindole method [26]. There are no pathology reports characterizing sympathetic catecholamine nerve loss following 6-OHDA. A report in MPTP-treated mice (30 mg/kg x 2 ip) showed 1 week after toxin delivery no qualitative change in TH and norepinephrine transporter (NET)-positive cardiac fibers and western blot analysis [40].

6-OHDA intoxication seemed to have a greater effect in myocardial sympathetic fibers than in nerve bundles. Sympathetic axons emerge from the cardiac ganglia and follow major arteries surrounding the left ventricle and collect in large epicardial or subepicardial bundles before extending into the myocardium as single fibers that eventually terminate onto cardiomyocytes. This organization implies that bundles in or near the epicardium contain axons closer to the sympathetic ganglia than myocardial fibers that are distal axons and closer to the terminal. This suggests that 6-OHDA is taken up mostly in the terminals of nerve fibers making them more susceptible to 6-OHDA-induced degeneration. A simpler explanation for differences between structures may be that the quantification method used for fibers compared to bundles had greater sensitivity to detect changes in sympathetic innervation. One PD study counted individual axons within cardiac nerve epicardial bundles [34], but our tissue did not allow for this quantification method since many of these structures exhibited intense immunostaining and individual axons were not easily identifiable.

The deficits in expression of TH and neuronal marker PGP9.5 three months after 6-OHDA dosing confirm neuronal loss. Down-regulation of catecholaminergic markers is a consequence of neurotoxins and additional markers are needed to assess true neurodegeneration. For example, quantification of nigral cells 1–4 days post-MPTP administration (20 mg/kg ip x 4) to mice induced a greater reduction in the number of TH positive nigral cells compared to Nissl-stained neurons [41].

Our study suggests that the percentage of TH expression per region in normal rhesus monkeys is similar to humans. The left ventricle of humans, nonhuman primates and other species is innervated by noradrenergic and cholinergic axons with varying regional distributions. In humans, TH-positive axons have been found more abundantly than AChE-positive axons in nerve bundles of the anterior (83%) compared to the inferior wall (28%) of the left ventricle [36]. Our results found a similar TH-positive distribution with control animals showing TH/PGP9.5 fiber area density of 84% in the anterior and 58% in the inferior wall. The remaining axons are possibly from parasympathetic innervation, however this was not evaluated in our study. Further investigation is necessary to understand if cholinergic nerves are affected by 6-OHDA treatment.

The differences between TH and PGP9.5 expression loss corroborate the specific sympathetic toxicity of 6-OHDA. This neurotoxin is selectively taken up by monoamine transporters and therefore its deleterious effects are limited to catecholaminergic neurons [21], which makes it an ideal agent to study the effects of sympathetic loss. To our knowledge there is no postmortem evidence of cholinergic nerve loss to the heart in PD patients. Interestingly, a report based on four patients with advanced PD that quantified neurofilament-positive epicardial nerve bundles in the anterior wall of the left ventricle described a 95% deficit of total axons compared to control patients [34], suggesting an effect on both sympathetic and parasympathetic innervation in the hearts of PD patients. However, authors did not conduct immunostaining for AChE-positive nerves. In PD, there is evidence of neuronal loss and Lewy pathologies in the dorsal motor nucleus of the vagus that has parasympathetic projections to the heart [42]. Functional clinical evidence of cardiac parasympathetic denervation has been reported with reductions in the high frequency spectral component of heart rate variability [43], [44], although parasympathetic contribution to this measure is controversial.

Dosing of 6-OHDA did not induce a pattern of neurodegeneration by level from the apex to the base or by layer from the subepicardium to the subendocardium of the left ventricle. Our data demonstrate an equal effect on TH-ir in fibers and nerve bundles across the apical to basal levels of the left ventricle. Human studies have shown greater TH-positive nerves in the base and epicardium of the left ventricle compared to the apex and the endocardium, respectively [36]. In PD, patterns across levels have not been evaluated by postmortem analysis, however, imaging studies have shown reduced MHED uptake in the more apical segments of the left ventricle lateral wall [45]. Diabetic patients have also shown impaired MHED uptake in nerves terminating near the apex, or the longest nerves in the heart [46], [47]. Sympathetic innervation of the heart across layers has not been investigated in PD.

Although there are few postmortem studies evaluating cardiac sympathetic denervation in toxin-induced models, several reports have used *in vivo* imaging [13]. In that regard, we recently reported that in these 6-OHDA-treated monkeys PET analysis at baseline compared to 14 weeks after 6-OHDA revealed a significant decrease in total (51%) MHED uptake in the left ventricle with greatest loss in the inferior (49.5% deficit) compared to the anterior (22.7% deficit) wall 3 months post-toxin [29]. Patterns of cardiac denervation have been found in PD using *in vivo* imaging, with the greatest loss localized to the inferior and lateral walls [25], [45], [48], [49], but this pattern has not been confirmed with postmortem analysis. In our monkeys comparison of *in vivo* MHED uptake and quantification of TH-ir in cardiac fibers across left ventricle walls showed significant correlations, suggesting left ventricle MHED uptake mirrors the amount of TH-positive nerve fibers. TH/PGP9.5 fiber area density ratios demonstrated significant loss in the inferior wall compared to the lateral wall in 6-OHDA monkeys, confirming that sympathetic innervation is mostly affected in the inferior wall 3 months after systemic administration of 6-OHDA. Analysis of TH-positive fibers or nerve bundles alone (not as a ratio to that of PGP9.5) in 6-OHDA monkeys showed no significant differences across walls. However, MHED is taken up by monoamine transporters and therefore it could be argued that immunostaining for anti-NET or anti-vesicular monoamine transporter 2 (VMAT2) would produce better matching results with MHED. Future investigation into the distribution of these proteins in systemic 6-OHDA-treated monkeys is warranted.

As MHED PET imaging in these monkeys showed some restoration of cardiac sympathetic innervation overtime, we evaluated measures of axonal recovery and found that the GAP43-positive nerve bundles score was increased in 6-OHDA-treated animals compared to controls [50]. Only a few axons within cardiac bundles co-expressed TH and GAP43, suggesting that most GAP43-ir axons were unable to exert autonomic influence on the heart. Regeneration of axons may lead to hyperinnervation of the myocardium, which has been tied to ventricular arrhythmias in heart transplant recipients [51]. We did not find increased PGP9.5 expression in the left ventricle, suggesting that hyperinnervation is unlikely at 3 months following 6-OHDA. *In vivo* evaluations for cardiac function using an echocardiogram and ECG in 6-OHDA-treated monkeys showed no signs of dysrhythmias, further suggesting the unlikely occurrence of hyperinnervation [29]. Studies with longer timelines are needed to understand if PGP9.5 or TH-immunoreactive fibers increase above baseline levels at timepoints greater than 3 months post-neurotoxin.

Markers of oxidative stress and inflammation were not upregulated in cardiac sections 3 months after systemic neurotoxin challenge. 6-OHDA induces catecholamine neuronal cell death by its auto-oxidation and further produces free radicals and increases generation of reactive oxygen species [52], [53], [54]. Intrastriatal administration of 6-OHDA to rats resulted in increased nitrotyrosine at 7 days post-lesion, yet the concentration of nitrotyrosine had significantly decreased compared to values quantified at 25 minutes after 6-OHDA [55]. Mice administered MPTP (10 mg/kg sc x 4), a dopaminergic neurotoxin, had increased nitrotyrosine expression in the substantia nigra 1 day after toxin challenge [56]. However, nitrotyrosine accumulation was not increased in the substantia nigra of monkeys 3 months after MPTP intoxication [57]. Similarly, inflammation has been observed in rats administered intrastriatal 6-OHDA where an inflammatory marker, Ox-6, was upregulated 32 days after toxin challenge [58]. *In vivo* imaging with PK11195 PET has also demonstrated a microglial response up to 4 weeks after intrastriatal 6-OHDA to rats [59]. There are no reports of upregulated inflammatory markers in the heart after systemic 6-OHDA treatment. These results from other animal studies suggest that the minimal changes in nitrotyrosine and HLA-DR expression after systemic 6-OHDA may be due to the timepoint of postmortem evaluation. In PD, the presence of reactive inflammatory cells immunostained for HLA-DR has been observed in the substantia nigra [60], [61]. To our knowledge there is no description of upregulated inflammatory markers in the heart of PD patients.

Neurodegenerative effects of 6-OHDA may account for the trend of reduced soluble α-synuclein-ir cardiac nerve bundles. The significant correlation between soluble α-synuclein scores and the AAT of TH-positive nerve bundles suggests soluble α-synuclein reflects the quantity of cardiac sympathetic axons. α-synuclein is a presynaptic protein associated to synaptic vesicles that is highly expressed in the brain [62], and has also been identified in peripheral tissues including the heart [63]. Under pathological conditions, α-synuclein aggregates form Lewy bodies [64], [65] and its phosphorylation further promotes accumulation and Lewy-related pathologies [66], [67]. We observed low amounts of phosphorylated α-synuclein that was unevenly expressed across groups. Mild expression may be typical as suggested by reports that found low amounts (4%) of α-synuclein phosphorylated at serine 129 in brains of normal rats [67]. In a single PD case report, Lewy axons were identified in epicardial nerve bundles using phosphorylated α-synuclein immunostaining [16]. Other studies have not identified phosphorylated α-synuclein in cardiac nerves of PD patients, but instead in the sympathetic ganglia in 80% of patients [68]. Interestingly, a recent report detected phosphorylated α-synuclein-ir in epicardial nerve bundles from biopsies of subjects that underwent cardiac surgery. Many of the subjects exhibited prodromal PD symptoms such as acting dreams and constipation, but no patient was diagnosed with PD at the time of the study. The authors suggest that follow-up studies of these patients are crucial to determine if patients will later be diagnosed with PD and to implicate the early detection of phosphorylated α-synuclein-ir in cardiac nerve bundles as a biomarker for PD [69].

Proteinase K resistant α-synuclein was absent from all animals. Reports of Lewy bodies with proven α-synuclein-positive intraneuronal inclusions as seen in PD patients have not been reported in toxin-models of PD. Instead MPTP-treated mice [70] and monkeys [71] have been shown to have upregulation of α-synuclein protein levels in the substantia nigra pars compacta and redistribution of α-synuclein-ir to the cell bodies of dopaminergic nigral neurons [70], [71], [72]. It is not clear if these changes precede cell death, and/or if the aggregation leading to typical Lewy bodies is not observed due to acute neurodegeneration and neuronal loss.

Adrenal medulla catecholaminergic deficits in 6-OHDA-treated monkeys resemble PD conditions. The function of the adrenal medulla is greatly compromised in PD and postmortem analysis has demonstrated a reduction of catecholamine markers [73], [74]. Interestingly, circulating catecholamines in PD have shown mixed results depending on the patient's condition during sample collection. Patients presenting orthostatic hypotension have reduced plasma norepinephrine compared to PD patients without intolerance [10], [11], [12]. Plasma catecholamine levels can also be affected by levodopa [75]. Our *in vivo* findings included decreased plasma catecholamines in the 6-OHDA monkeys, which matched the decreased expression of TH-ir and AADC-ir in chromaffin cells. Circulating 6-OHDA can be taken up into NET in the plasma membrane of chromaffin cells [76]. Although earlier studies in mice have investigated catecholamines in the adrenal medulla, TH immunostained adrenal tissue has not been assessed following 6-OHDA intoxication [77].

The similar expression of PGP9.5 in the adrenal medulla of control and 6-OHDA-treated monkeys suggests that the chromaffin cells are dysfunctional but otherwise intact. PGP9.5 is a neural enzyme involved in the processing of ubiquinated proteins. PGP9.5-ir was first identified in neuroendocrine cells [78], [79] and chromaffin cells of the adrenal medulla are derived from embryonic neural crest and often termed adrenal paraneurons and share similar proteins as postganglionic nerves. Similar characterization of PGP9.5-ir in the adrenal medulla has been described in rats [80] and cotton-top tamarins [81]. Quantification of TOMM20-ir did not show any changes post-6-OHDA, suggesting that catecholamine dysfunction in chromaffin cells is not due to an effect in mitochondrial activity at this timepoint.

α-synuclein soluble, phosphorylated, or PK resistant did not show changes in the adrenal medulla of 6-OHDA compared to control monkeys. In PD, Lewy bodies have been observed in ganglia within the adrenal medulla and periadrenal fatty tissue [18], [19], [82]. Phosphorylated α-synuclein-ir fibers have been observed as punctate or diffuse staining in the adrenal medulla of PD patients [68]. Few control animals expressed high amounts of soluble α-synuclein with patches of proteinase K resistant α-synuclein, yet the exact cellular location of α-synuclein is not clear. Soluble α-synuclein resembled cross sections of axons as seen in heart. However, PGP9.5-ir of the adrenal medulla did not match soluble α-synuclein expression, suggesting that it may not be located in nerves. Other studies have described α-synuclein expression in Golgi apparatus of bovine adrenal medulla [83]. Further analysis is required to clearly identify the cellular location of soluble α-synuclein in the adrenal medulla.

In summary, this postmortem analysis indicates systemic administration of 6-OHDA to rhesus monkeys produces cardiac neurodegeneration and adrenal catecholaminergic dysfunction and confirms our previous *in vivo* data. The obtention of normal tissues from WNPRC tissue bank is an example of reduction of animal use. This is the first report in nonhuman primates to quantify, in detail, cardiac sympathetic innervation after neurotoxin challenge. Our novel method to quantify catecholamine fiber area density is sensitive and could be applied to estimate fiber area density in cardiac tissue of animal models of PD and PD patients. Our results also suggest that the nonhuman primate model of cardiac dysautonomia induced by systemic 6-OHDA is a valid model to test neuroprotective strategies and determine their efficacy using *in vivo* and postmortem outcome measures.
